# Long-Term Traumatic and Asymptomatic Aorto-Right Atrial Fistula

**DOI:** 10.21470/1678-9741-2018-0296

**Published:** 2019

**Authors:** Sandro Gonçalves de Lima, Andréa Bezerra de Melo da Silveira Lordsleem, Kaique Leal Campos, Juliana Guedes Silva, Marcelo Antônio Oliveira Santos, Brivaldo Markman Filho

**Affiliations:** 1 Department of Cardiology, Hospital das Clínicas at Universidade Federal de Pernambuco (HC-UFPE), Recife, PE, Brazil.; 2 Epidemiology and Cardiology Research Group, Recife, PE, Brazil.

**Keywords:** Fistula/Surgery, Heart Atria, Wounds, Penetrating, Wounds and Injuries, Heart Injuries

## Abstract

Aorto-atrial fistulas due to cardiac trauma are rare, and survivors require immediate surgical correction. Here, we report a case of an aorto-right atrial fistula due to penetrating trauma after a 16-year evolution, which developed symptoms of acute coronary syndrome and was treated with myocardial revascularization and correction of the aorto-cameral fistula.

**Table:** 

Abbreviations, acronyms & symbols	
ARAF	= Aorta-right atrial fistula
RA	= Right atrium
TEE	= Transesophageal echocardiography

## INTRODUCTION

The aorta-right atrial fistula (ARAF) is a rare aortic-chamber connection that may originate from one of the three sinuses of Valsalva. Traumatic cause is rare and commonly associated with the need for emergency surgical correction^[1]^.

Victims of cardiac trauma who have survived intracardiac lesions such as ventricular septal defect, ARAF and aorta-ventricle fistula, with sufficient time to undergo corrective surgery are rare^[2]^.

We report a case of ARAF after a penetrating thoracic trauma due to a cold weapon after 16 years of evolution.

### Clinical Data

A 66-year-old male, hypertensive, smoker, reported epigastric pain, irradiated to the mandible, associated with sweating two years before admission. Four months before admission he had presented paroxysmal nocturnal dyspnea, orthopnea, lower limb edema, asthenia, visual turbidity and dizziness. A previous history of precordial trauma due to a cold weapon, 16 years before, was treated only with pleural drainage.

In the physical examination, a scar was observed on the right parasternal line at the level of the fifth intercostal space, due to the cold weapon injury, jugular turgor at 90º, mesocardial impulsion with no thrill. Regular two-stroke heart rhythm with continuous 3+/6+ murmur in the accessory aortic and aortic focus, and hyperphoneme components A2 and P2.

### Electrocardiography

**Fig. 1:**
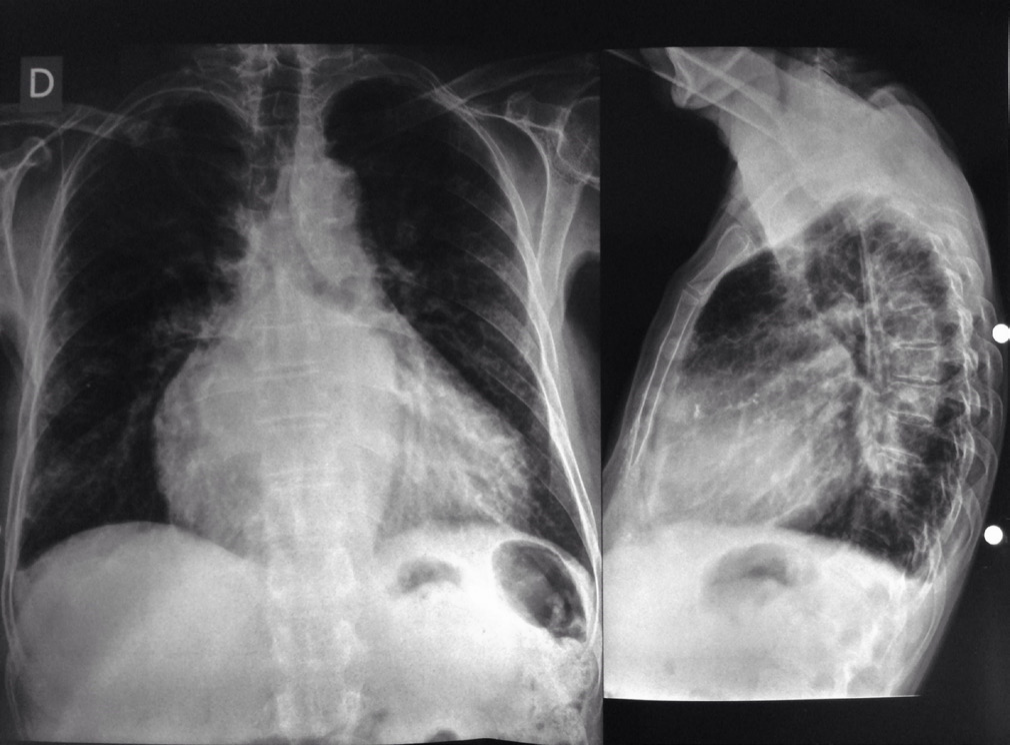
Posterior-anterior (left) and profile (right) chest radiography.

First-degree atrioventricular block. Signs of left ventricular overload and slow R wave growth in the anterolateral wall (Figure 1).

### Radiography

**Fig. 2:**
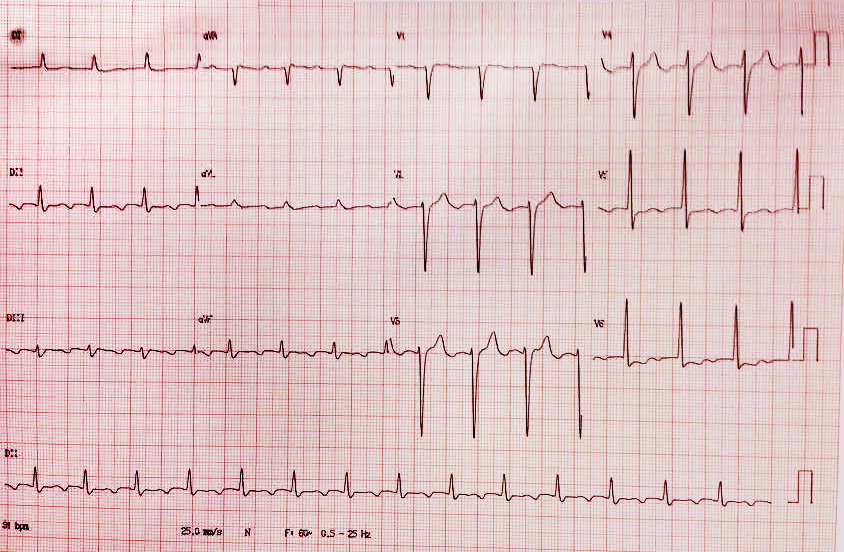
Electrocardiogram.

Increased cardiac chambers, especially right and left atria, and increased pulmonary vascular markings (Figure 2).

### Transthoracic Echocardiography

Left ventricular ejection fraction of 41.1%, septal hypokinesia, right chamber dilatation, right ventricle with diffuse hypocontratility (tricuspid annular plane systolic excursion = 15 mm), and systolic and diastolic flow of the right coronary aortic sinus into the right atrium (RA).

### Diagnosis and Surgery

**Fig. 3:**
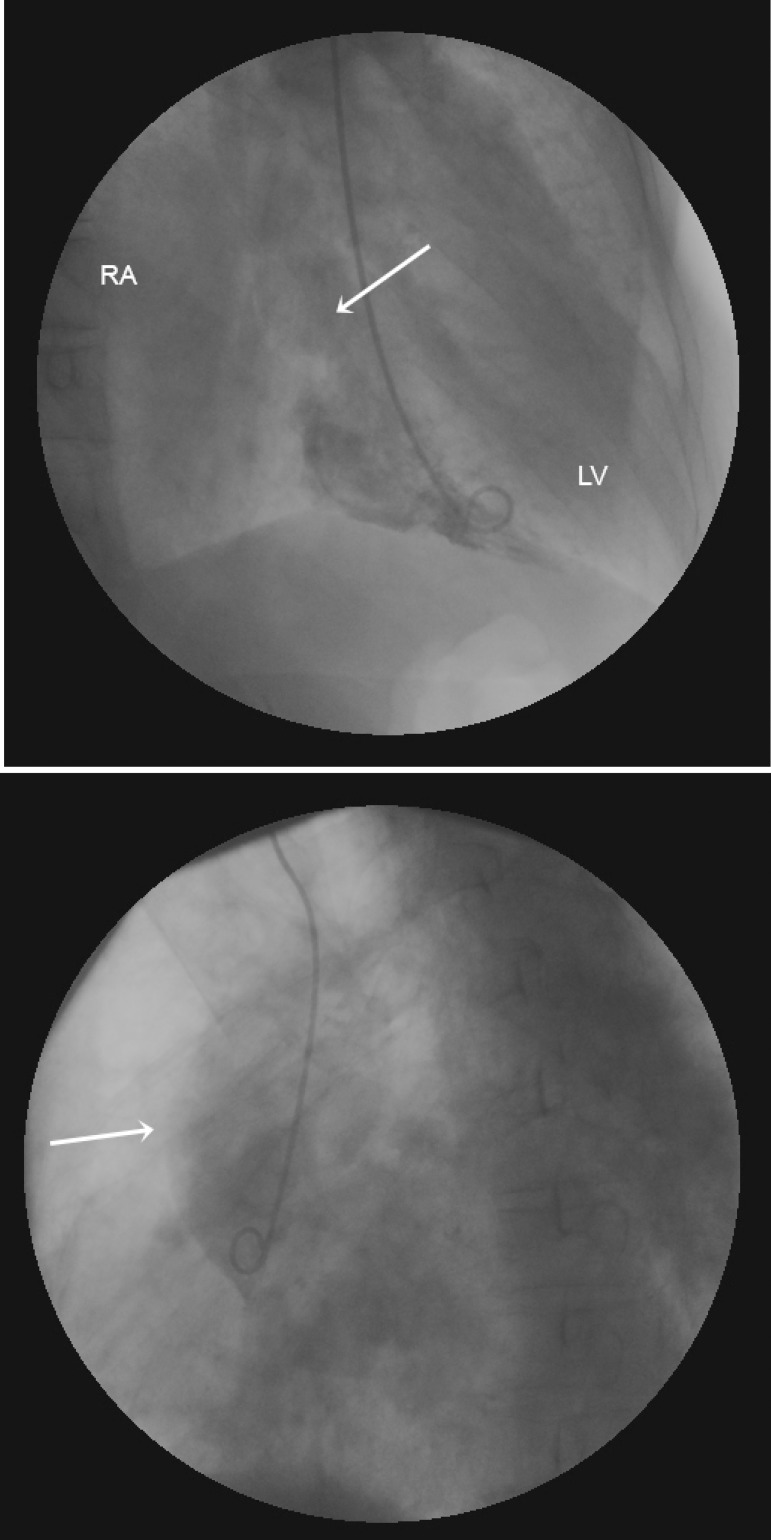
Ascending aorta aortography: blood flow (white arrow) from the aorta to the right atrium (RA). LV=left ventricle

Cineangiocoronariography demonstrated a severe distal lesion in the trunk of the left coronary artery, anterior descending with severe proximal lesion, circumflex occluded in the distal third and right coronary with no significant lesions (Movie 1). In aortography, the ascending aorta exhibited a preserved caliber, a right ventral sinus rupture image with consequent formation of ARAF (Figure 3, Movies 2 and 3).

**Video 1:**
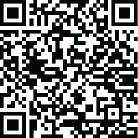
Cineangiocoronariography.

**Video 2:**
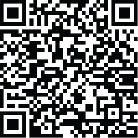
Aortography of the ascending aorta.

**Video 3:**
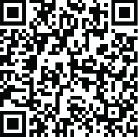
Aortography of the ascending aorta.

The patient underwent surgical correction of the fistula by direct suture and myocardial revascularization (left internal thoracic artery-anterior descending, saphenous vein graft from the aorta to two marginal branches), with good evolution.

## DISCUSSION

We have encountered only one report of ARAF through trauma from a cold weapon, described by Swanepoel et al.^[3]^. However, in this case, the patient presented with symptoms of rapidly progressive heart failure in the first four days after the trauma. Stable evolution over a period as long as 16 years, as presented by our patient, demonstrates the rarity of the case herein described.

In patients with asymptomatic aorto-chamber fistula, clinical findings depend on the affected chamber. The presence of a continuous murmur appears to be present in most cases of ARAF described, as well as in ours^[3-6]^. In general terms, diagnosis is possible through transesophageal echocardiography (TEE), which demonstrated a communication between the flow from the aorta to the cardiac chamber. However, cardiac angiography is the gold standard for the diagnosis of fistula and is indicated when TEE is inconclusive.

Early surgical repair is recommended as the treatment of choice, given the need to prevent the development of symptoms and serious complications such as ventricular overload, bacterial endocarditis, pulmonary vascular disease, aneurysm formation and the risk of spontaneous rupture^[7,8]^. The closure of the ARAF by transcatheter device has been considered an innovative method and may be used as an alternative to the traditional surgical approach in selected cases^[8]^.

**Table:** 

Authors' roles & responsibilities	
SGL	Conception and study design; manuscript redaction or critical review of its content; final manuscript approval
ABMSL	Conception and study design; manuscript redaction or critical review of its content; final manuscript approval
KLC	Manuscript redaction or critical review of its content; final manuscript approval
JGS	Manuscript redaction or critical review of its content; final manuscript approval
MAOS	Manuscript redaction or critical review of its content; final manuscript approval
BMF	Manuscript redaction or critical review of its content; final manuscript approval
